# The relationship between allergic rhinitis and sleep disorders and mental health

**DOI:** 10.3389/falgy.2026.1806555

**Published:** 2026-04-10

**Authors:** Xiaoning Feng, Zhaowei Gu, Yunxiu Wang

**Affiliations:** 1 Department of Clinical Epidemiology, Clinical Research Center, Shengjing Hospital of China Medical University, Shenyang, China; 2 Department of Otolaryngology Head and Neck Surgery, Shengjing Hospital of China Medical University, Shenyang, China; 3 Department of Clinical Trial Ward, Clinical Trial and Conversion Center, Shengjing Hospital of China Medical University, Shenyang, China; 4 Department of Pharmaceutical Toxicology, School of Pharmacy, China Medical University, Shenyang, China

**Keywords:** allergic rhinitis, anxiety, depression, HPA axis, mental health, sleep quality

## Abstract

Allergic Rhinitis (AR) is a highly prevalent IgE-mediated chronic inflammatory disease of the nasal mucosa worldwide. Its incidence is continuously increasing and has become one of the important chronic diseases affecting public health. Recent studies have gradually revealed that AR not only presents with local symptoms such as nasal congestion, runny nose, sneezing, and nasal itching, but is also closely related to a wide range of physical and mental health problems, especially mental and psychological disorders (such as anxiety and depression) and decreased sleep quality. There is a complex two-way or even circular interaction between these three factors. This article systematically reviews the epidemiological characteristics and pathophysiological mechanisms of AR, and focuses on exploring the association pathways and potential mechanisms between AR and mental health as well as sleep disorders, including neuroimmune regulation (such as inflammatory mediators, HPA axis function), psychological and behavioral factors, and social function impairment. Based on this, the article further summarizes the assessment tools and multimodal intervention strategies for mental and psychological problems and sleep disorders in AR patients, covering drug treatment, psychotherapy, sleep hygiene education, and comprehensive health management, etc. This article integrates current evidence and provides a theoretical framework for a comprehensive understanding of the multi-dimensional impact of AR, and offers references for comprehensive assessment and individualized intervention in clinical practice.

## Introduction

## 1 Global burden, symptom classification, clinical manifestations, and pathogenesis of allergic rhinitis

### 1.1 Global burden of allergic rhinitis

AR ranks among the most common chronic diseases worldwide, with its prevalence increasing notably—especially in developed countries. The World Health Organization estimates there are approximately 400–600 million AR patients globally, affecting about 10%–30% of adults and up to 40% of children (1). Prevalence varies significantly across countries and regions, generally being higher in industrialized nations and urban areas, and showing an overall upward trend. It is also one of the most common chronic diseases among children and adolescents. In Asia, AR affects around 9% of the population, compared to 14% in the United States and 7% in Latin America (2). In Europe, the prevalence of AR among Danish adults has steadily increased over the past three decades, rising from 19% to 32% (3). Similarly, the standardized prevalence of AR among Chinese adults has increased by 6.5% over the past six years (4); in the grassland regions of Northern China, due to extremely high seasonal pollen concentrations, the self-reported prevalence of pollen-induced allergic rhinitis (PiAR) has reached a peak of 32.4% (5). Three national cross-sectional surveys conducted among Japanese children between 2005 and 2015 also revealed a continuous increase in rhinoconjunctivitis cases.

**Table 1:** Key research summaries on the relationship between allergic rhinitis and sleep quality and mental health.

First Author & Year	Study Design	Sample Size	Study Population Characteristics	Assessment Tools	Key Findings
Léger et al. (17)	Cross-sectional	2,018	AR patients and healthy controls	Questionnaire	Approximately 40%–60% of AR patients had sleep quality problems, significantly higher than controls
Rodrigues et al. (13)	Systematic review and meta-analysis	10 studies	AR patients	HADS, SCL-90, etc.	AR patients had significantly higher risks of anxiety and depression compared to healthy controls
Liu et al. (34)	Systematic review and meta-analysis	16 studies	AR patients	PSQI, ESS, etc.	AR was significantly associated with sleep disorders, with a mean difference in PSQI scores of 2.1 points
Koinis-Mitchell et al. (39)	Review	N/A	Children with AR	PSG, questionnaires	Children with AR exhibit disrupted sleep architecture, which may affect daytime function and cognitive performance
Thompson etal. (38)	Clinical study	35	Patients with moderate-to-severe AR	PSG, inflammatory markers	Slow-wave sleep reduced by 38.5 min, correlated with IL-1β levels
Safia et al. (20)	Meta-analysis	8 studies	AR patients	Psychological assessment scales	Significantly higher prevalence of mental health problems (anxiety/depression) in AR patients
Høj et al. (50)	Systematic review and meta-analysis	15 studies	AR patients	Suicidal ideation assessment	AR patients had significantly higher risk of suicidal ideation compared to healthy controls
Rosenkranz et al. (41)	Functional MRI study	20	AR patients (pollen season vs. non-pollen season)	fMRI, inflammatory markers	Activation in the insula and anterior cingulate cortex increased by 32.7% during pollen season in AR patients
Nakamura et al. (37)	Animal study	Mouse model	Ovalbumin-sensitized mice	Gene expression, behavioral tests	Sensitized mice exhibited disrupted circadian expression rhythms of Clock genes in the suprachiasmatic nucleus and fragmented sleep-wake cycles

It is worth noting that the epidemiological characteristics of allergic rhinitis (AR) vary considerably across different regions. Environmental factors play a critical role in both the onset and exacerbation of the disease. For example, in colder regions, prolonged exposure to indoor allergens such as dust mites and molds is common due to extended indoor living (6), while in dry and windy seasons, the dispersion of airborne allergens like pollen is significantly increased (7). In addition, air pollutants can directly irritate the respiratory mucosa and also act as carriers that adsorb allergens, enhancing their allergenicity and tissue penetration, thereby aggravating AR symptoms and airway inflammation (8). Indoor allergens—including dust mites, molds, pet dander, and cockroaches—are the primary triggers of perennial AR (9, 10), whereas seasonal pollen from plants such as Artemisia, Humulus, Ragweed, Poplar, Willow, and Elm contributes to the prevalence of seasonal AR (7).

In Northeast China, the unique geographical climate and social environment have shaped distinct epidemiological features of AR. The region experiences long, cold winters with an extended heating season lasting approximately five to six months. During this period, residents tend to spend more time in sealed indoor environments, leading to increased exposure to indoor allergens such as dust mites, mold, pet dander, and cockroaches. This contributes to a higher prevalence of perennial AR and potentially more severe symptoms (11). Furthermore, the substantial indoor-outdoor temperature difference in winter, along with the irritant effect of cold air itself, acts as a significant non-specific trigger that can induce or worsen nasal symptoms.

Although epidemiological surveys on AR prevalence and allergen sensitization profiles have been conducted in several areas, comprehensive and systematic regional data remain limited (12). Currently, there is insufficient research exploring how specific environmental determinants—such as cold exposure, air pollution, and mixed indoor-outdoor allergen interactions—affect AR pathogenesis, clinical presentation, disease severity, and treatment outcomes. These gaps underscore the need for future region-specific investigations.

Beyond its clinical manifestations, AR imposes a substantial burden on both individuals and society. Direct medical costs include expenses related to consultations, diagnostic tests, medications, and surgical interventions. However, the indirect costs—often overlooked—are equally significant, particularly those arising from sleep disturbances and associated mental health conditions. These include reduced work productivity, absenteeism, impaired learning efficiency, school absenteeism, and diminished quality of life. Evidence suggests that the economic impact of daytime dysfunction and reduced performance due to AR-related sleep problems may even exceed direct medical expenditures (13). In regions such as Europe and North America, the total economic burden of AR on society and individuals amounts to hundreds of billions or even trillions of dollars annually. Therefore, a comprehensive understanding of the multidimensional health impacts of AR is of great public health importance. Early diagnosis, standardized treatment, and long-term management are essential to mitigate this growing burden.

### 1.2 Symptom classification and clinical manifestations of allergic rhinitis

AR can be classified by symptom duration into intermittent and persistent types, and by symptom severity and impact on quality of life into mild and moderate-severe types (14). This classification standard was recently revised by the Allergic Rhinitis and its Impact on Asthma (ARIA) workshop (15).

Nasal congestion is the core symptom most closely associated with sleep disorders. Nighttime nasal congestion leads to breathing difficulties, mouth breathing, and frequent awakenings, directly affecting the sleep architecture and recovery functions (16). Studies have shown that sleep disorders in patients with moderate to severe allergic rhinitis (AR) manifest as difficulty falling asleep, fragmented sleep, reduced deep sleep, and shortened rapid eye movement (REM) sleep (17). Long-term nighttime nasal congestion can induce or exacerbate obstructive sleep apnea, forming a vicious cycle of “nasal congestion—OSA—more severe nasal congestion” (16, 18).

Sudden symptoms such as sneezing, runny nose, and itchy nose are closely related to psychological stress. Frequent nose-blowing, eye-rubbing, and nose-rubbing behaviors may cause social embarrassment, especially among children and teenagers, which can lead to ridicule or isolation from peers, causing patients to feel shy and exhibit social withdrawal behaviors (19–22). This impairment of social function can directly trigger anxiety and affect mental health.

The close comorbidity relationship between AR and asthma further exacerbates the burden on the body and mind. Approximately 40% of AR patients have asthma as a co-morbidity (23), and asthma itself is an independent risk factor for sleep disorders and emotional problems, making AR patients vulnerable to physical and mental health issues.

### 1.3 Pathogenesis of allergic rhinitis

AR is a chronic inflammatory disease of the nasal mucosa mediated by immunoglobulin E (24). The core mechanism of its pathogenesis is a Th2-type immune response: in individuals with atopy, upon exposure to allergens, specific IgE antibodies are produced, sensitizing mast cells and basophils; upon re-exposure to the same allergen, the allergen binds to IgE, triggering these cells to release various inflammatory mediators such as histamine, leukotrienes, and cytokines (25).This immune response process can be divided into two stages: the sensitization stage, where the body produces specific IgE antibodies upon its first exposure to the allergen, and these antibodies bind to the surface of mast cells, putting the body in a sensitized state (23, 26–28); the effector stage, when the same allergen is encountered again, the allergen binds to IgE, triggering the release of granules from mast cells, and releasing pre-synthesized and newly generated inflammatory mediators, thereby initiating the immediate phase and delayed phase reactions (29–31).

Recent studies have confirmed that these inflammatory mediators not only act locally but also can affect the central nervous system through various pathways, forming a neural-immune bridge connecting AR with sleep disorders and mental health problems (32, 33). This mechanism constitutes the core theoretical framework of this review:

Firstly, the inflammatory cytokines in the circulation can enter the central nervous system. The continuously elevated inflammatory cytokines (such as IL-1β, IL-6, TNF-α) in AR patients can act on brain regions related to emotion and sleep regulation (such as the hippocampus, amygdala, and hypothalamus) through active transport across the blood-brain barrier or the vagus nerve input pathway (32). These factors can inhibit hippocampal neurogenesis, activate the amygdala, and increase the susceptibility to anxiety and depression (22, 33).

Secondly, inflammatory factors can directly interfere with sleep regulation. IL-6 and TNF-α can inhibit the synthesis and secretion of melatonin by the pineal gland. Clinical studies have shown that the peak level of serum melatonin in AR patients at night is 27.3% lower than that of healthy controls, and it is negatively correlated with the PSQI score (34).

Animal experiments have confirmed that egg white-sensitized mice have disrupted circadian rhythm expression of the Clock gene in the suprachiasmatic nucleus, and their sleep-wake cycle is fragmented (35).

Thirdly, inflammatory factors affect sleep structure. Polysomnography studies have shown that patients with moderate to severe AR have a reduction of 38.5 ± 6.2 min in slow-wave sleep time and an increase of 22.4 ± 5.1 min in the latency of rapid eye movement sleep (36). This change is correlated with an increase in serum IL-1β levels (*r* = 0.51), suggesting that inflammation may cause a decrease in sleep depth through the HPA axis activation (37). Additionally, IL-6 levels are negatively correlated with sleep spindle density (*r* = −0.39), indicating that inflammation may affect the function of the thalamus-cortex circuit (37).

Fourth, the neuro-sensitization mechanism plays a significant role in AR-related sleep disorders. Functional magnetic resonance imaging studies have found that the activation of the insular cortex and the anterior cingulate gyrus in AR patients during the pollen season was 32.7% higher than in non-allergic seasons (38). Histological studies have shown that the density of sensory nerve fibers in the nasal mucosa of AR patients increased by 1.8 times, and the expression of TRPV1 receptors was upregulated. This may be the anatomical basis for the enhanced sensitivity to nocturnal nasal stimuli (39).

It is worth noting that sleep deprivation itself can further exacerbate the inflammatory response, creating a two-way vicious cycle. Experimental sleep restriction (4 h per day for 5 days) increased the serum IL-6 level in AR patients by 41.5% and the TNF-α level by 29.3% (40). Recent clinical studies have found that anti-IL-4Rα monoclonal antibody treatment can increase the sleep efficiency of AR patients by 18.7 ± 3.2% (41), further confirming the core role of the inflammatory mechanism in AR-related sleep disorders. This “inflammation—sleep disorder” two-way interaction constitutes the pathological physiological basis for the persistent and aggravation of physical and mental symptoms in AR patients.

### 1.4 Disease burden and related comorbidities of allergic rhinitis

The most important comorbidity of AR is bronchial asthma (23, 42). From the core theme of this review, asthma itself is an independent risk factor for sleep disorders and emotional problems, which makes patients with AR and asthma require special attention to their sleep quality and mental health status (11).

Among other related comorbidities, the bidirectional relationship between obstructive sleep apnea and AR is particularly worthy of attention (16, 18): On one hand, nasal congestion increases nasal resistance, forcing one to breathe through the mouth, which can trigger or exacerbate OSA; on the other hand, the chronic inflammation associated with AR can spread to the upper airway soft tissues, causing lymphoid tissue hyperplasia and mucosal edema in the pharynx, resulting in physical narrowing of the upper airway. And the intermittent hypoxia caused by OSA can activate the systemic inflammatory response, mutually promoting the local inflammation of AR and forming a vicious cycle (Figure 4).

Furthermore, comorbidities such as chronic rhinosinusitis (43, 44) and atopic dermatitis (11) have further exacerbated the overall sleep burden and psychological stress of the patients.

AR is an IgE-mediated chronic inflammatory disease of the nasal mucosa, with a continuously rising prevalence globally and in China. Influenced by factors such as long cold winters, dominant indoor exposure during the heating period, specific pollen seasons, and air pollution, Northeast China exhibits unique epidemiological and clinical characteristics—with dust mites as the main perennial allergen, Artemisia and other pollens as important seasonal allergens, and the region being affected by composite environmental exposure (pollution + cold). AR poses challenges to both individual health and socioeconomic well-being. Therefore, early diagnosis, standardized treatment, and long-term management are crucial. Future research should focus on obtaining precise local data, deeply analyzing the pathogenic mechanisms of composite environmental exposures, and exploring optimized diagnosis, treatment, and prevention strategies adapted to the special environment of Northeast China to reduce the disease burden of AR in this region.

## 2 Search strategy

This article is a narrative review aimed at integrating existing evidence and exploring the relationship between allergic rhinitis and sleep quality as well as mental health. To cover the relevant literature as comprehensively as possible, we conducted a systematic search of the following electronic databases in October 2025: PubMed, Web of Science, Scopus, and PsycINFO. The search strategy combined subject terms and free words, and the core search formula was as follows: (“allergic rhinitis” OR “hay fever” OR “rhinoconjunctivitis”) AND (“sleep” OR “insomnia” OR “sleep quality” OR “PSQI” OR “sleep disorder”) AND (“mental health” OR “depression” OR “anxiety” OR “psychiatry” OR “HADS” OR “SCL-90”). The search time range was from January 2000 to September 2025. Inclusion criteria were: (1) original research or systematic review; (2) the research subjects were humans; (3) the core content involving the relationship between AR and sleep and/or mental health; (4) published in English or Chinese. Exclusion criteria included case reports, conference abstracts, editorials, and non-peer-reviewed literature. We conducted an in-depth manual screening of the references of the included studies to supplement potential missing literature. This review focused on integrating high-quality evidence from cross-sectional studies, longitudinal cohort studies, meta-analyses, and mechanistic studies (Table 1).

## 3 The relationship between allergic rhinitis, mental health, and sleep quality

### 3.1 Allergic rhinitis and mental health

In recent years, the negative impact of AR on patients' mental health has garnered increasing attention. Numerous clinical observations and epidemiological studies indicate that AR patients have a higher incidence of emotional disorders such as anxiety and depression (45). Rodrigues et al. conducted a systematic review and meta-analysis of 10 studies, which confirmed that patients with AR had significantly higher risks of anxiety and depression compared to healthy controls, and this association was more prominent in patients with moderate to severe AR (13). Cross-sectional studies based on the SCL-90 scale also found that the anxiety and depression factor scores of AR patients were significantly higher than those of healthy individuals, and the psychological symptoms were more obvious in patients with moderate to severe AR (19).

**Figure 1:**
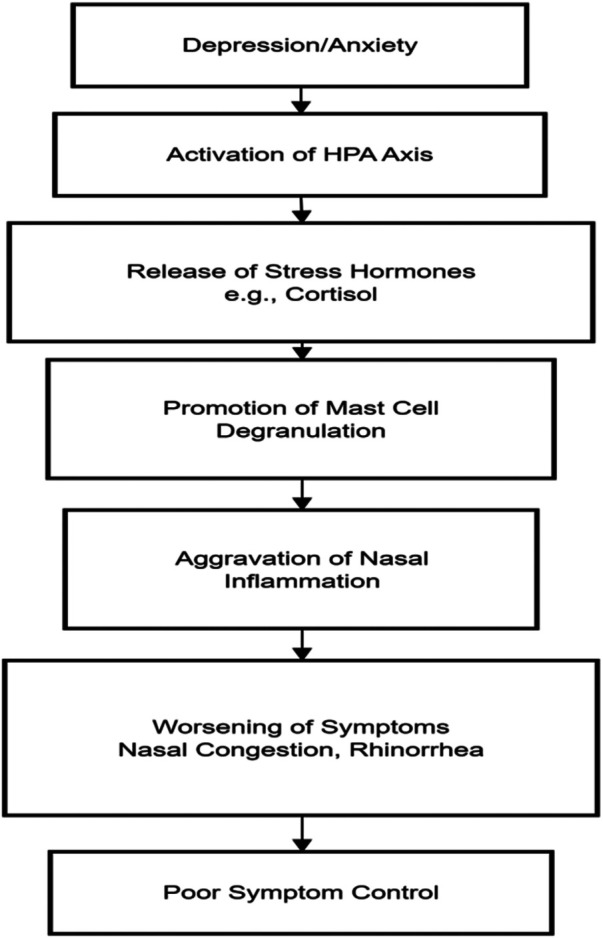
Pathways of the impact of mental health on allergic rhinitis.

Illness perception in AR patients is a key mediating factor affecting their psychological state. Illness perception refers to the patient's cognitive representation and emotional response to their illness, including their understanding of the illness identity (symptom label), timeline (chronic/acute nature), consequences, personal control, and causes (46). Research shows that AR patients with low self-efficacy regarding disease control—i.e., those who believe they cannot manage their symptoms—have significantly higher levels of anxiety and depression (47). This feeling of “loss of control” may stem from the unpredictability of symptoms (e.g., sudden sneezing) and dissatisfaction with treatment outcomes, forming a vicious cycle: “symptom onset → negative cognition (“I can't control this”) → emotional distress (anxiety/depression) → heightened symptom perception (attentional fixation)”. Conversely, patients with high self-efficacy are more likely to adhere to treatment and adopt active coping strategies (e.g., avoiding allergens), thereby better controlling symptoms and maintaining psychological stability (48). As shown in Figure 1.

Nasal symptoms in AR patients—especially persistent nasal congestion—can significantly reduce quality of life, thereby inducing or exacerbating psychological problems. Nasal congestion causes difficulty breathing at night, affecting sleep quality. Chronic sleep deprivation impairs emotional regulation ability, increasing the risk of anxiety and depression (13). Furthermore, frequent sneezing and runny nose can make patients feel embarrassed in social situations, even leading to social avoidance behaviors. A cross-sectional study based on the SCL-90 scale found that the anxiety and depression factor scores of AR patients were significantly higher than those of healthy populations, with moderate-severe AR patients showing more prominent psychological symptoms (19). Persistent nasal symptoms (especially stubborn nasal congestion) not only cause physical discomfort but also lead to impaired social functioning, restricted daily activities, reduced work or study efficiency, and even social avoidance behaviors, thereby inducing or worsening negative emotional states.

This impact is particularly prominent and specific in children and adolescent AR patients (20). This group is in a critical period of psychological and behavioral development, and the distress caused by AR often translates into externalizing behavioral problems. Nocturnal nasal congestion and sleep fragmentation directly lead to daytime sleepiness, poor concentration, and memory decline, severely affecting their academic performance (21). Meanwhile, due to frequent nose wiping, eye rubbing, and nose rubbing, children may face teasing or isolation from peers, developing feelings of shame and social withdrawal, and becoming unwilling to participate in group activities (e.g., physical education, outdoor play), which affects the normal development of their social skills. In the long term, this may lead to low self-esteem and loneliness, laying the groundwork for mental health issues in adulthood (22).

More alarmingly, recent large-sample studies and meta-analyses have begun to reveal a potential association between AR and extreme psychological outcomes—suicidal ideation and behavior. A systematic review and meta-analysis indicated that the risk of suicidal ideation in AR patients was significantly higher compared to healthy controls. The potential mechanisms are multifaceted: first, persistent pro-inflammatory cytokines (e.g., IL-6, TNF-α) in a state of chronic inflammation may directly affect the brain, impacting neural circuits related to mood regulation and impulse control; second, long-term sleep deprivation, daytime functional impairment, chronic pain (e.g., headaches), and potential accompanying economic burden collectively constitute significant psychological stressors; finally, as mentioned earlier, comorbid depressive/anxiety disorders are themselves major risk factors for suicide. Although a direct causal relationship between AR and suicidal behavior requires further confirmation, this finding strongly suggests the importance of mental health screening and risk assessment for moderate-severe or refractory AR patients in clinical practice.

#### 3.1.1 Mechanisms by which allergic rhinitis affects mental health

The pathogenesis of AR involves a Th2-type immune response, accompanied by the release of cytokines such as IL-4, IL-5, and IL-13. Recent research has found that these inflammatory factors not only act on the local nasal mucosa but may also affect the central nervous system via the blood-brain barrier or vagus nerve signaling. Chronic immune activation and inflammatory cytokine release (e.g., IL-6, TNF-α) in a persistent allergic state are also believed to potentially affect neurotransmitter balance through the “brain-immune axis,” directly participating in the pathophysiology of mood regulation disorders.

**Figure 2:**
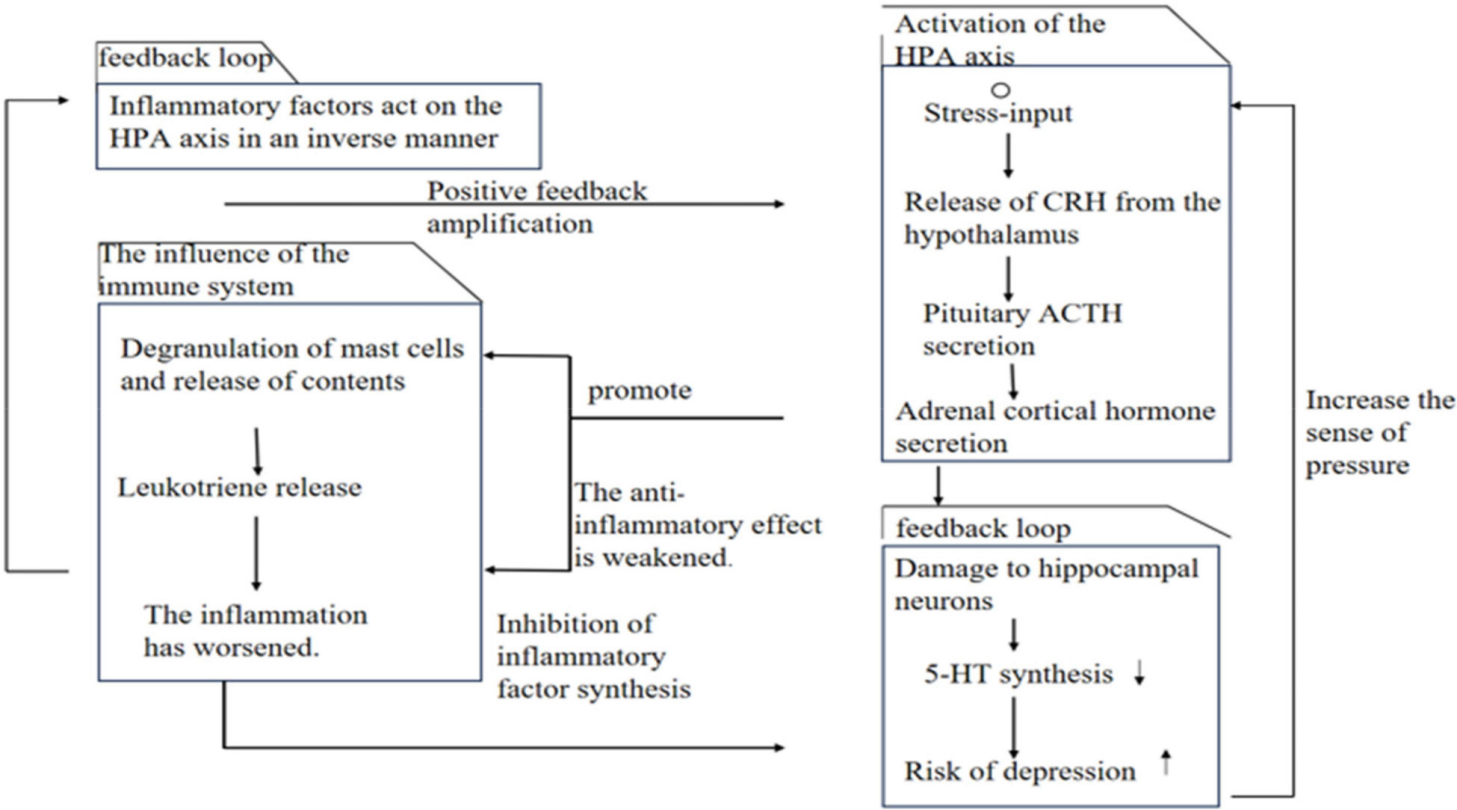
Pathways of the impact of mental health on allergic rhinitis.

Persistently elevated inflammatory cytokines (e.g., IL-1β, IL-6, TNF-α) in AR patients can affect the central nervous system via the blood-brain barrier or vagus nerve afferent signals. These factors can inhibit hippocampal neurogenesis (associated with depression) (22) and activate the amygdala (the emotional fear center), increasing susceptibility to anxiety and depression. Depression/anxiety activates the HPA axis, releasing stress hormones such as cortisol, which then promote mast cell degranulation, leading to worsened nasal inflammation and symptoms (e.g., nasal congestion, rhinorrhea). Depressed patients are more likely to miss medications (e.g., intranasal corticosteroids), leading to poor symptom control and forming a vicious cycle (49), as shown in Figure 2.

#### 3.1.2 Limitations and controversies of existing studies

Although numerous studies have confirmed the association between AR and mental health problems, the existing evidence still has certain limitations. Firstly, most studies are cross-sectional in design, making it difficult to determine the causal relationship—is AR causing mental problems, or are mental problems exacerbating AR symptoms? Secondly, different studies use various assessment tools (SCL-90, HADS, BDI, etc.), making it difficult to directly compare the results. Thirdly, regarding the differences in mental health risks among patients with different AR phenotypes (intermittent/continuous, mild/moderate/severe), current research is insufficient and more stratified analyses are needed. Fourthly, although the potential association between AR and suicidal ideation has been suggested (50), the direct causal relationship between the two still needs to be further confirmed through well-designed prospective studies.

#### 3.1.3 Tools for assessing mental health

The Symptom Checklist-90 (SCL-90) is a commonly used mental health assessment tool. It contains 90 items, each rated on a 1–5 scale, covering 10 psychological factors. The total average score is calculated as the total score of 90 items divided by 90; factor scores are calculated as the total score of items for each factor divided by the number of items in that factor. Symptom classification criteria are as follows: a factor score and total average score <1.5 indicates no symptoms; ≥1.5 and <2.5 indicates mild symptoms; ≥2.5 and <3.5 indicates mild-moderate symptoms; ≥3.5 and <4.5 indicates moderate-severe symptoms; and ≥4.5 indicates severe symptoms (50).

Activation of the HPA axis feedback loop; Inflammatory factors act on the HPA axis in an inverse manner; Stress-input; Positive feedback amplification; Release of CRH from the hypothalamus; The influence of the immune system; Pituitary ACTH secretion; Degranulation of mast cells and release of contents; Promote; Adrenal cortical hormone secretion; Increase the sense of pressure; Leukotriene release; The anti-inflammatory effect is weakened; Damage to hippocampal neurons; The inflammation has worsened; Inhibition of inflammatory factor synthesis; 5-HT synthesis; Risk of depression).

### 3.2 Allergic rhinitis and sleep quality

AR patients often experience varying degrees of sleep disturbance due to nasal symptoms, involving multiple mechanisms including physiological, neuroimmune, and behavioral aspects (17). Studies show that approximately 40%–60% of AR patients have sleep quality issues, mainly manifesting as difficulty falling asleep, increased nighttime awakenings, disrupted sleep architecture, and daytime sleepiness. In-depth analysis of the mechanisms by which AR affects sleep helps develop more precise clinical intervention strategies.

For pediatric patients, the impact of AR on sleep architecture is particularly profound and may have long-term consequences for growth, development, and cognition. Growth hormone is primarily secreted in pulses during deep sleep (i.e., slow-wave sleep), and the sleep architecture of AR children is characterized by reduced and fragmented slow-wave sleep (51). This may potentially limit growth hormone secretion, theoretically having a negative impact on children's height growth. Furthermore, sleep—especially REM sleep—is crucial for memory consolidation and learning. Frequent nighttime awakenings and disrupted sleep architecture caused by AR, particularly the shortening of REM sleep, severely interfere with the consolidation of declarative and procedural memory (52). This provides a key pathophysiological basis for explaining why AR children often experience learning difficulties and memory decline.

Nasal congestion, as a core symptom of AR, often leads to difficulty breathing at night, mouth breathing, frequent awakenings, and can even induce or worsen Obstructive Sleep Apnea (OSA) (16). The disruption of sleep architecture manifests as difficulty falling asleep, sleep fragmentation, reduced deep sleep, and shortened REM sleep, directly affecting the restorative function of sleep.

The consequences of this sleep disturbance are directly reflected in multiple key areas of daytime function. Excessive daytime sleepiness and poor concentration are the most common daytime manifestations in AR patients. In student populations, this directly translates to decreased academic performance, with absenteeism also rising due to symptoms and fatigue. In working populations, reduced work efficiency, slowed reactions, and impaired decision-making due to sleep deprivation constitute a huge indirect economic burden. More alarmingly, there is a public safety risk: some studies suggest that uncontrolled AR patients, due to poor sleep quality leading to daytime sleepiness, have a significantly increased risk of driving accidents, comparable to that of patients with mild sleep apnea.

The comorbidity between AR and Obstructive Sleep Apnea has become a research hotspot, with a significant bidirectional aggravating relationship. The comorbid mechanism is complex and bidirectional: on one hand, nasal congestion increases nasal resistance, forcing mouth breathing, which can cause the tongue to fall back and the upper airway to collapse, thereby inducing or worsening OSA (18); on the other hand, the chronic inflammation associated with AR itself can spread to the soft tissues of the upper airway. Inflammatory mediators (e.g., leukotrienes, histamine) can cause hyperplasia of pharyngeal lymphoid tissue (e.g., adenoids, tonsils) and edema of the pharyngeal mucosa, thereby physically narrowing the upper airway caliber. Additionally, intermittent hypoxia and sleep fragmentation caused by OSA are themselves stressors that can further activate systemic inflammatory responses, forming a vicious cycle with the local inflammation of AR, leading the two conditions to mutually promote and worsen. Long-term sleep disturbance not only exacerbates daytime fatigue, poor concentration, and cognitive decline but also forms a vicious cycle of “symptoms—poor sleep—worsened mood—heightened symptom perception,” further damaging the patient's physical and mental health (53). Structural equation modeling confirmed that the PSQI mediated 42% (95% CI: 35%–49%) of the effect between AR and depression.

#### 3.2.1 Mechanisms by which allergic rhinitis affects sleep quality

Sleep disturbance in AR patients is closely related to Th2-type inflammatory responses. Studies show that levels of inflammatory cytokines such as IL-4, IL-5, IL-6, IL-13, and TNF-α are significantly elevated in the nasal mucosa of AR patients, and these cytokines can interfere with sleep regulation through various pathways (44).

Regarding circadian rhythm regulation, IL-6 and TNF-α can inhibit the synthesis and secretion of melatonin by the pineal gland. Clinical studies show that the nocturnal peak serum melatonin level in AR patients is 27.3% lower than in healthy controls (*p* < 0.01), and it is negatively correlated with PSQI scores (*r* = −0.42 (34). Animal experiments confirm that ovalbumin-sensitized mice exhibit disrupted circadian expression rhythms of Clock genes in the suprachiasmatic nucleus and fragmented sleep-wake cycle (35).

The impact of inflammatory factors on sleep architecture is particularly significant. PSG studies show that moderate-severe AR patients have 38.5 ± 6.2 min less slow-wave sleep time (*p* < 0.001) and a 22.4 ± 5.1-min prolongation of REM sleep latency (*p* < 0.01) (36). This change is correlated with elevated serum IL-1β levels (*r* = 0.51), possibly due to HPA axis activation leading to reduced sleep depth (37). Furthermore, IL-6 levels are negatively correlated with sleep spindle density (*r* = −0.39, *p* < 0.05), suggesting inflammation may affect thalamocortical circuit function (37).

Neural sensitization mechanisms play an important role in AR sleep disturbance. fMRI studies found that activation in the insula and anterior cingulate cortex of AR patients during pollen season increased by 32.7% compared to the non-allergy season (*p* < 0.001) (38). Histological studies show that the density of sensory nerve fibers in the nasal mucosa of AR patients increases 1.8-fold, and TRPV1 receptor expression is upregulated, which may be the anatomical basis for enhanced nocturnal nasal irritation sensitivity (39).

Notably, sleep deprivation can further aggravate the inflammatory response. Experimental sleep restriction (4 h/day × 5 days) increased serum IL-6 levels by 41.5% (*p* < 0.01) and TNF-α by 29.3% (*p* < 0.05) in AR patients (40). This bidirectional interaction forms a vicious cycle of “inflammation-sleep disturbance.” Recent clinical studies found that anti-IL-4Rα monoclonal antibody treatment improved sleep efficiency by 18.7 ± 3.2% in AR patients (*p* < 0.001), but long-term effects still require further observation (41).

#### 3.2.2 Limitations and controversies of existing studies

Although progress has been made in mechanism research, the existing evidence still has certain limitations. Firstly, most mechanism studies are based on small samples or animal experiments and need to be verified in large population groups. Secondly, the severity and phenotype of AR patients in different studies may affect the results, but there is currently a lack of stratified analysis. Thirdly, for the long-term effects of sleep disorders in children with AR (such as their impact on cognitive development and academic performance), more longitudinal studies are still needed. Fourthly, although anti-inflammatory treatments (such as anti-IL-4Rα monoclonal antibodies) show the potential to improve sleep (41), their long-term effects and safety still need further observation.

### 3.2.3 Sleep quality assessment tools

Currently, various common assessment tools are available to measure the degree of sleep quality impairment in patients affected by AR (54). Disease-specific quality of life questionnaires (such as the Rhinoconjunctivitis Quality of Life Questionnaire, RQLQ) are widely used to assess the impact of the disease on health, and many of these questionnaires include sleep-related questions (55, 56). In clinical trials, generic and disease-specific quality of life questionnaires combined with symptom assessment have been used to obtain information on drug efficacy (57). Sleep quality analysis reveals some characteristics of AR that cannot be detected through daytime symptom assessment alone (58). Available objective measures include polysomnography, multiple sleep latency test, maintenance of wakefulness test, and learning and performance tests (39), which can help assess the impact of AR on sleep.

### 3.3 The interaction between allergic rhinitis, sleep quality, and mental health

There is a close bidirectional relationship between mental health status and sleep quality. Psychological stress and emotional disorders themselves can lead to insomnia or poor sleep quality; conversely, sleep deprivation or disrupted sleep architecture are proven to be important risk factors for inducing anxiety and depression. In AR patients, this complex relationship may be significantly amplified: rhinitis symptoms act simultaneously as a “stressor” and a “sleep disruptor,” while psychological distress and sleep disorders may reduce the patient's ability to cope with symptoms and treatment adherence, forming a pathological network intertwined with multiple factors.

Neuroimmune regulation plays a central role in this triangular relationship. Elevated pro-inflammatory cytokines like IL-6 and TNF-α in AR patients can affect central nervous system function via the blood-brain barrier or vagus nerve afferent pathways (32). These inflammatory factors can inhibit hippocampal neurogenesis while activating the amygdala, leading to emotional regulation disorders and changes in sleep architecture (33). Clinical studies show that serum IL-6 levels in AR patients are significantly positively correlated with both PSQI scores (*r* = 0.48) and HADS depression scores (*r* = 0.52) (*p* < 0.001) (33).

Abnormal activation of the HPA axis is another key link. Psychological stress can promote cortisol release, and cortisol can enhance mast cell degranulation, worsening nasal inflammation (59). A 6-month longitudinal study found that AR patients with worsening depressive symptoms had a 37.5% increase in histamine levels in nasal secretions compared to baseline (*p* < 0.01), along with a 2.3-fold increase in nighttime awakenings (60).

**Figure 3:**
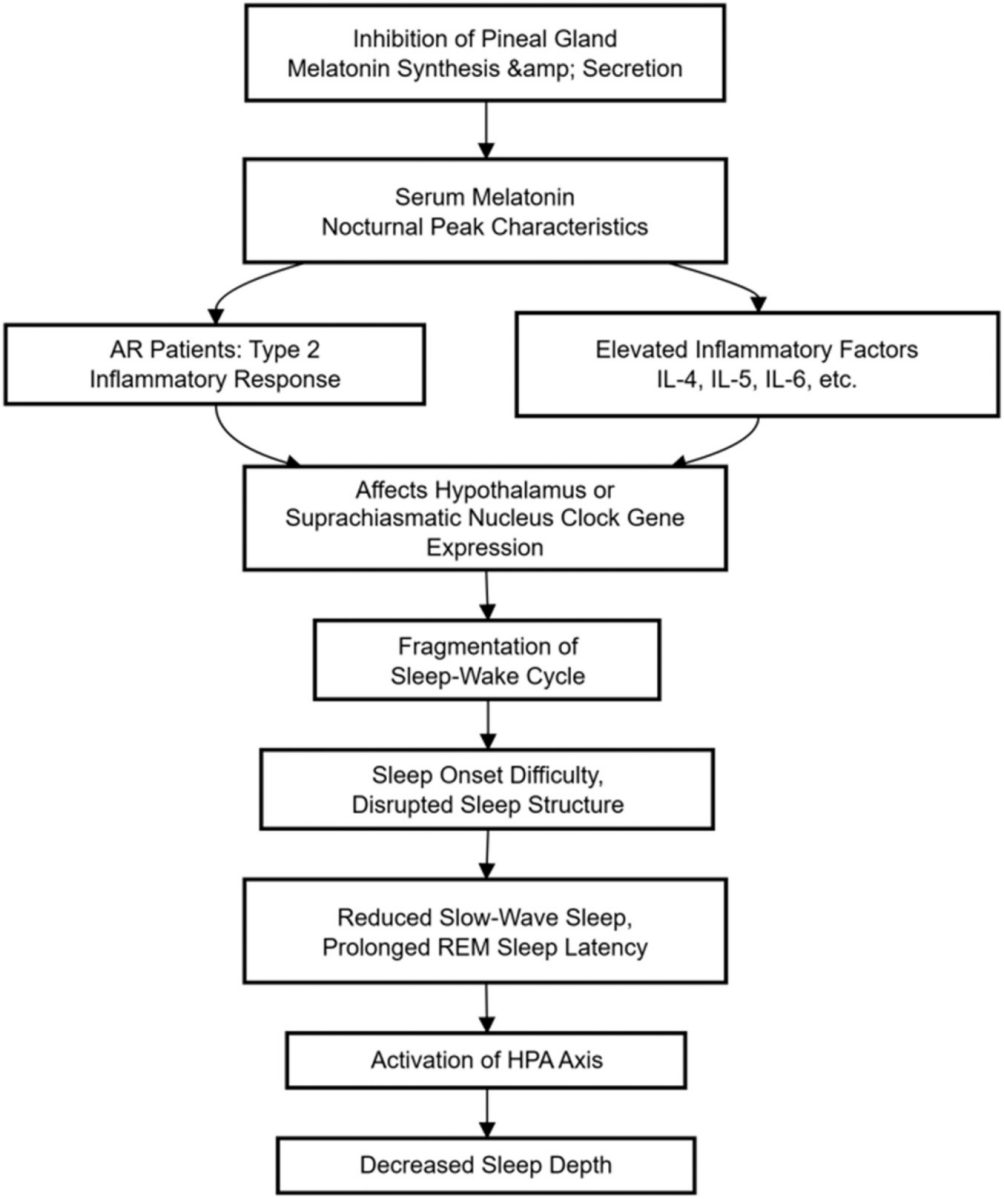
The mechanism by which allergic rhinitis affects sleep quality.

Sleep disturbance significantly amplifies the negative impact of AR on mental health. PSG studies confirmed that for every 1-h reduction in slow-wave sleep time in AR patients, negative emotion scores the next day increased by 18.7% (*p* < 0.01) (61). This effect is particularly prominent in adolescent populations; the incidence of depression in AR adolescents with sleep disturbance is 2.1 times that of AR patients without sleep disturbance (95% CI: 1.6–2.8) (62). Sleep disturbance amplifies psychological and physiological damage through a cascade effect of decreased sleep quality: sleep deprivation inhibits prefrontal function leading to decreased emotional regulation ability (irritability, anxiety); REM sleep reduction hinders emotional memory processing, leading to accumulation of negative emotions (increased depression risk); circadian rhythm disruption leads to HPA axis dysregulation (abnormal cortisol rhythm); ultimately, this worsens AR symptoms.As shown in Figure 3.

**Figure 4:**
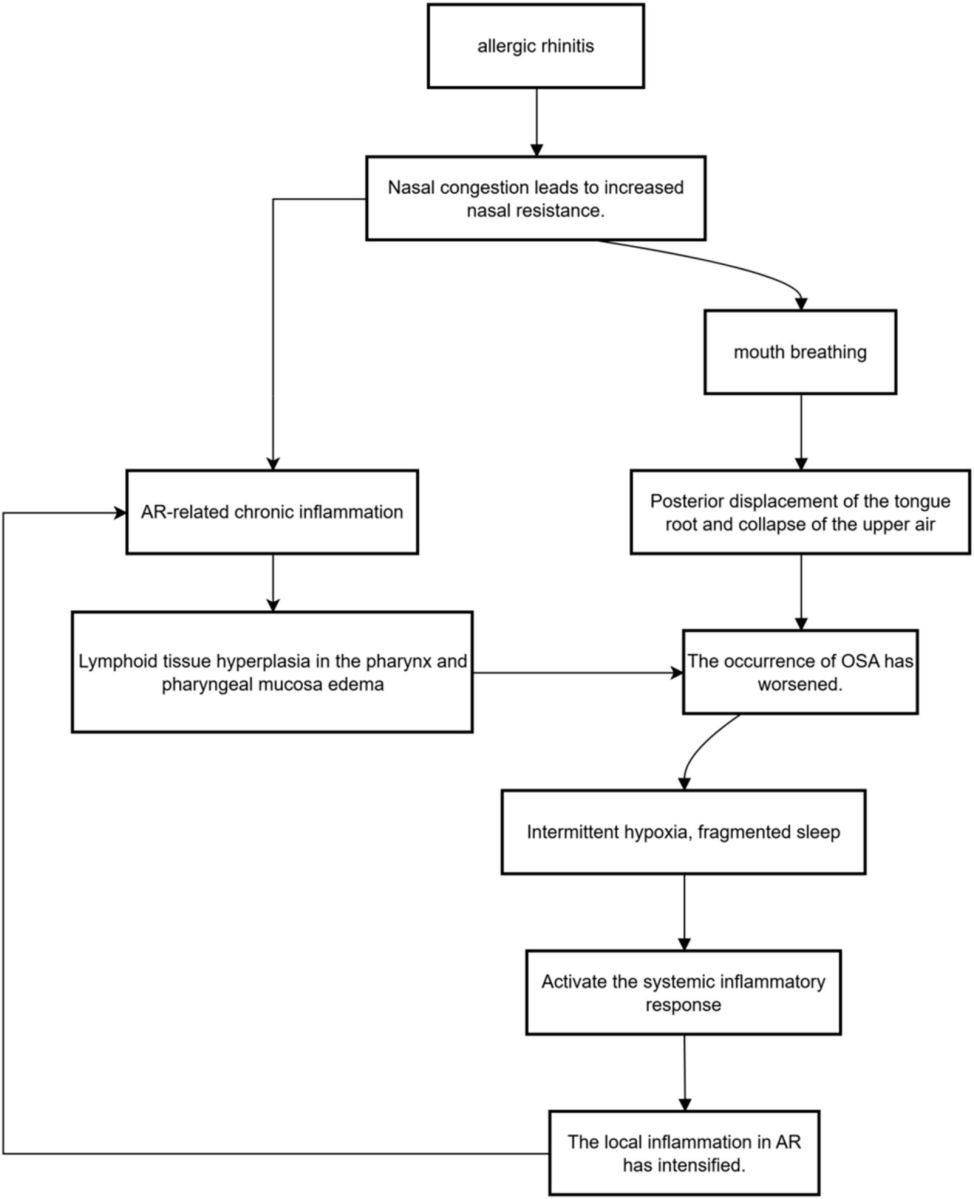
Bidirectional interactions between allergic rhinitis and obstructive sleep apnea (OSA).

The purpose of understanding the “inflammation-sleep-mood” vicious cycle is to find breakthroughs for clinical intervention. The key to breaking this cycle lies in multi-target intervention. First, effective control of nasal inflammation is the cornerstone (63). Standardized use of intranasal corticosteroids not only alleviates local symptoms but the reduction in systemic inflammation levels may also positively affect mood and sleep. Second, directly targeting HPA axis dysfunction and circadian rhythm disruption—such as supplementing exogenous melatonin for patients with insufficient melatonin secretion—has been shown to simultaneously improve sleep latency, regulate immune responses, and potentially reduce neuroinflammation through its antioxidant properties. Furthermore, psychological interventions like mindfulness-based stress reduction and cognitive-behavioral therapy to modulate stress responses can lower cortisol levels, thereby reducing its activating effect on mast cells, inhibiting physiological inflammation from the psychological side (64).

Psychological interventions, especially Cognitive Behavioral Therapy (CBT), show unique value in breaking this vicious cycle. Cognitive Behavioral Therapy for Insomnia (CBT-I) can help AR patients correct unreasonable beliefs about sleep (e.g., “I must get 8 h of sleep to work”), reduce sleep effort, and establish regular sleep-wake rhythms (64). Research shows that CBT-I not only improves sleep in patients with primary insomnia but also significantly alleviates their comorbid anxiety and depression symptoms. Applying its principles to AR patients can not only directly improve sleep disturbances caused by symptoms but also, by enhancing patients' emotional regulation ability and sense of control over symptoms (self-efficacy), indirectly reduce excessive attention to and catastrophic thinking about nasal symptoms, thus forming a positive feedback loop. CBT focused on disease management can teach patients to better cope with the life restrictions and social embarrassment caused by AR, reducing psychological stress at the source.

On the other hand, effective treatment of comorbid sleep disorders can also positively feedback on AR symptoms and psychological state (65). For patients with AR comorbid with Obstructive Sleep Apnea (OSA), Continuous Positive Airway Pressure (CPAP) therapy, while improving hypoxia and sleep architecture, can also significantly reduce morning nasal congestion and daytime sleepiness. The mechanism may be that the physical pressure provided by CPAP supports the collapsed upper airway, reducing the nasal congestion feedback loop caused by mouth breathing; simultaneously, by restoring normal sleep architecture, CPAP helps reset the disrupted HPA axis and immune rhythms, thereby systemically reducing the inflammatory load. As mentioned earlier, melatonin supplementation can target sleep disorders, immune dysregulation, and HPA axis dysfunction simultaneously from the perspective of regulating the biological clock, offering new ideas for the comprehensive management of AR., as shown in Figure 4.

## 4 Clinical management strategies for sleep and mental health issues in patients with allergic rhinitis

### 4.1 The gap between research and practice

Although numerous high-quality studies have confirmed the close relationship between allergic rhinitis and sleep quality as well as mental health, there are still significant challenges in translating these research findings into routine clinical practice in otolaryngology. This section aims to systematically analyze this “evidence-practice” gap and provide clinical practitioners with practical and feasible initial screening and management strategies.

#### 4.1.1 Limitations of professional knowledge

The core expertise of otolaryngologists lies in the organic and inflammatory diseases of the upper airway. They lack systematic training in the diagnosis and treatment of mental and psychological disorders, and it is not a core concern in their daily practice. When dealing with an AR patient who may have anxiety or depression, clinicians often encounter the following difficulties: being able to perceive the problem but unable to define it, being able to define the problem but unable to handle it, and being afraid to “open Pandora's box” and thus not daring to ask questions proactively.

#### 4.1.2 The realistic challenges of referral

Theoretically, referring patients suspected of having psychological problems to the psychiatry or psychology departments is the best approach. However, in practice, there are numerous obstacles: patient perception issues—in many cultural contexts, especially in East Asian societies such as China, mental health problems still face severe “stigmatization”. Patients may have a strong aversion to “visiting a psychologist” and believe that “I'm not crazy, why go to a psychology department?”; medical resource and cost issues—there is a shortage of qualified psychological counselors, the waiting time after referral is long, and most psychological counseling is not covered by medical insurance, resulting in additional financial burdens that deter patients; poor referral coordination—there is a lack of effective communication channels between otolaryngology doctors and psychology doctors, leading to fragmented patient management.

### 4.2 Tailored practical outpatient tools for otolaryngologists

In light of the aforementioned challenges, otolaryngologists require a streamlined, efficient, and outpatient-friendly assessment tool. The goal is not to make psychiatric diagnoses, but to conduct effective “risk stratification” and “initial screening” to determine whether the next step is “soothing observation”, “strengthening AR treatment”, or “recommending specialist consultation”.

We recommend a “2 + 1” simple outpatient tool package, which can conduct rapid screenings for sleep and psychological issues within 3–5 min. It can be operated and interpreted without the need for a background in psychology.

Using the core question format, the following four dimensions are mainly inquired about: sleep onset time (how long does it usually take to fall asleep), nocturnal awakenings (whether one wakes up due to nasal congestion or breathing difficulties), snoring/obstructed breathing (whether others have reported snoring or whether one feels breathing pauses themselves), daytime sleepiness (whether one feels sleepy and lacks energy during the day). A total score >5 indicates significant sleep quality issues.

Action Plan: If the PSQI score is abnormal, the first step should be to review the AR control situation—have sufficient nasal corticosteroids been used? Has the nasal congestion been effectively controlled? If after 4–8 weeks of intensified AR treatment, the sleep quality still does not improve, or the PSQI score is extremely high (>10 points), or the patient has obvious snoring or apnea symptoms, the possibility of coexisting obstructive sleep apnea should be considered. A STOP-Bang questionnaire screening or referral to a sleep center should be conducted.

The HADS was specifically designed for non-psychiatric outpatient departments in general hospitals. Its greatest advantage lies in eliminating the items that might be confused with physical symptoms, allowing for a purer assessment of the core emotions of anxiety and depression. The entire questionnaire consists of only 14 questions, divided into two sub-scales: anxiety (HADS-A) and depression (HADS-D). The completion time is approximately 3–5 min. Scoring criteria: 0–7 points indicate normal, 8–10 points indicate mild abnormality, 11–14 points indicate moderate abnormality, and 15–21 points indicate severe abnormality.

Action Plan: Mild abnormalities (HADS score 8–10) may be directly related to uncontrolled rhinitis symptoms. A strategy of “intensified AR treatment followed by a follow-up visit after 4 weeks” can be adopted. Moderate to severe abnormalities (HADS score ≥11) or those with suicidal ideation mentioned in the questionnaire must be referred. Communication skills are crucial during the referral process—doctors need to “normalize” the referral suggestion, comparing it to “visiting a cardiologist for hypertension”, in order to reduce the patient's sense of stigma: “Your current emotional state requires professional help. It's the same as seeing a cardiologist for hypertension. The psychology department has some very effective treatment methods that can make your overall condition improve faster.”

### 4.3 Establishing a “patient-centered” comprehensive management mindset

Upgrade of routine consultation: During each follow-up visit, in addition to asking about nasal symptoms, two additional questions should be routinely included: “Have you been sleeping well at night recently? Do you wake up due to nasal congestion?” “Have you felt irritable or depressed recently because of nasal inflammation?” These two simple questions, combined with the occasional use of the aforementioned questionnaire, can significantly enhance the understanding of the patient's overall health condition without significantly increasing the burden on the outpatient department.

Re-definition of successful treatment: The traditional criterion for the success of AR treatment mainly focused on the improvement of nasal symptoms. In future clinical practice, the definition of successful treatment should be expanded to include multiple dimensions of outcomes—control of nasal symptoms, improvement of sleep quality, stability of emotional state, and restoration of social function.

Collaborative network construction: It is suggested that hospitals or departments with the conditions should actively establish referral collaboration networks with psychology departments, psychiatry departments, and sleep medicine centers. They should clearly define the referral indications and procedures, regularly conduct multidisciplinary case discussions, jointly formulate integrated treatment paths, and provide patients with a “one-stop” integrated management experience.

## 5 Management and future perspectives for allergic rhinitis with sleep and mental health issues

### 5.1 Optimization and validation of multidisciplinary integrated intervention models

Based on an in-depth understanding of the complex relationship between AR and mental health and sleep quality, clinical practice and public health strategies need to shift from simply controlling nasal symptoms to a multidimensional integrated management model (66).

Routine screening for psychological and sleep problems should be implemented: incorporate SCL-90 depression/anxiety factors, PSQI, or HADS scales into the routine assessment of AR patients (especially those with moderate-severe, persistent cases). AR is a global disease with significant health and socioeconomic burden (39, 67). Fully recognizing its multidimensional impact beyond the nose—particularly the close association with mental health disorders (anxiety, depression) and decreased sleep quality, and the complex bidirectional, vicious cycle mechanisms—is crucial for improving overall patient prognosis (68).

In clinical practice, assessment of psychological state and sleep quality should be incorporated into the routine management of AR (especially moderate-severe cases), and integrated intervention strategies should be adopted: optimize nasal symptom control (paying special attention to nasal congestion management), identify and address coexisting anxiety, depression, and sleep disorders, strengthen patient education and environmental control (in Northeast China, special attention should be paid to indoor allergens, air pollution, and cold stimuli (69).

### 5.2 In-depth exploration of neuroimmune mechanisms and targeted therapies

#### 5.2.1 Central role of inflammatory factors

Study the specific pathways through which inflammatory factors like IL-6 and TNF-α affect mood and sleep via the blood-brain barrier (70), and explore the potential of anti-inflammatory drugs (e.g., IL-4Rα monoclonal antibodies) to improve psychological symptoms.

#### 5.2.2 HPA axis regulation

Develop intervention measures targeting Hypothalamic-Pituitary-Adrenal (HPA) axis dysregulation, such as using melatonin supplementation or stress management training (e.g., mindfulness therapy) to alleviate cortisol rhythm abnormalities in AR patients (71).

### 5.3 Future perspectives on allergic rhinitis with mental health or sleep quality issues

Although existing studies have separately explored the relationship between AR and mental health or sleep quality, research systematically analyzing their interaction mechanisms and comprehensive impact within a unified framework remains relatively insufficient (72).

Especially among AR patients with different severity levels and phenotypes (intermittent/persistent), whether differences exist in this association, and whether psychological interventions or sleep improvement measures can serve as adjuvant treatment strategies to enhance the overall efficacy of AR, these questions still require in-depth exploration (17). Therefore, this study aims to comprehensively investigate the mental health status (anxiety, depression levels) and sleep quality status of AR patients, analyze the correlation between AR and psychological/sleep indicators, and explore the interaction pathways among the three. The research results will provide evidence for understanding the multidimensional health burden of AR and provide a theoretical basis for developing comprehensive intervention strategies integrating nasal symptom control, psychological support, and sleep management, ultimately improving patients' overall quality of life and long-term prognosis (20).

Future research should focus on validating the effectiveness of multidisciplinary integrated intervention models, exploring new therapies like neuroimmune targeted treatments and neuromodulation, and deeply analyzing disease characteristics in the unique Northeastern environment to develop precise prevention and control strategies. Only through comprehensive, individualized management can the vicious cycle of “rhinitis-sleep-mood” be effectively broken, significantly enhancing the quality of life and long-term health outcomes of AR patients (73).

## 6 Conclusion

Allergic rhinitis, as a common chronic inflammatory disease worldwide, has a far-reaching impact on patients beyond the local symptoms of the nasal cavity. This article systematically reviews the existing evidence and reveals the complex and closely interrelated bidirectional interaction between AR and sleep quality as well as mental health: Core symptoms such as nasal congestion directly disrupt the sleep structure through mechanical obstruction; Inflammatory mediators affect the central nervous system through neuro-immune pathways, interfering with emotional regulation and circadian rhythms; Psychological distress and social function impairment further exacerbate symptom perception and treatment difficulties, forming a vicious cycle of mutual reinforcement.

This understanding holds significant guiding significance for clinical practice. Otolaryngologists should incorporate sleep quality and mental health status into the routine assessment of AR, use simple tools such as the PSQI core questions and HADS for rapid screening, and implement stratified management based on the screening results—for those with mild abnormalities, strengthen AR treatment; for those with moderate to severe abnormalities, promptly refer them to a psychology department or sleep center. At the same time, through communication skills for “normalization” of referral suggestions, it can effectively reduce patients' sense of stigma and improve the acceptance of referrals.

Future research needs to further clarify the precise mechanisms of neuro-immune regulation, conduct large-sample prospective studies to verify causal relationships, explore individualized treatment strategies based on biomarkers, and pay attention to the influence of environmental factors in specific regions (such as Northeast China) on the multi-dimensional health outcomes of AR. Only by integrating a comprehensive management model that combines nasal symptom control, sleep improvement, and psychological support can we truly break the vicious cycle of “rhinitis—sleep—mood”, and comprehensively improve the quality of life and long-term health outcomes of AR patients.

## References

[1] Yin Leung AS Tham EH Samuel M Munblit D Chu DK Dahdah L Quality and consistency of clinical practice guidelines on the prevention of food allergy and atopic dermatitis: systematic review protocol. World Allergy Organ J. (2022) 15(9):100679. 10.1016/j.waojou.2022.100679 36185546 PMC9478906

[2] Meltzer EO Blaiss MS Naclerio RM Stoloff SW Derebery MJ Nelson HS Burden of allergic rhinitis: allergies in America, Latin America, and Asia-Pacific adult surveys. Allergy Asthma Proc. (2012) 33(Suppl 1):S113–41. 10.2500/aap.2012.33.3603 22981425

[3] Leth-Møller KB Skaaby T Linneberg A. Allergic rhinitis and allergic sensitisation are still increasing among Danish adults. Allergy. (2020) 75(3):660–8. 10.1111/all.14046 31512253

[4] Wang XD Zheng M Lou HF Zhang Y Bo MY Ge SQ An increased prevalence of self-reported allergic rhinitis in major Chinese cities from 2005 to 2011. Allergy. (2016) 71(8):1170–80. 10.1111/all.12874 26948849 PMC5074323

[5] Ma T Wang X Zhuang Y Shi H Ning H Zhang T Prevalence and risk factors for allergic rhinitis in adults and children living in different grassland regions of inner Mongolia. Allergy. (2020) 75(1):234–9. 10.1111/all.13941 31169905

[6] Bousquet J Van Cauwenberge P Khaltaev N, Aria Workshop Group, World Health Organization. Allergic rhinitis and its impact on asthma. J Allergy Clin Immunol. (2001) 108(5 Suppl):S147–334. 10.1067/mai.2001.118891 11707753

[7] Maurer M Zuberbier T. Undertreatment of rhinitis symptoms in Europe: findings from a cross-sectional questionnaire survey. Allergy. (2007) 62(9):1057–63. 10.1111/j.1398-9995.2007.01367.x 17581263

[8] Durham SR. The inflammatory nature of allergic disease. Clin Exp Allergy. (1998) 28(Suppl 6):20–4. 10.1046/j.1365-2222.1998.0280s6020.x 9988430

[9] Skoner DP. Allergic rhinitis: definition, epidemiology, pathophysiology, detection, and diagnosis. J Allergy Clin Immunol. (2001) 108(1 Suppl):S2–8. 10.1067/mai.2001.115569 11449200

[10] Wallace DV Dykewicz MS Bernstein DI Blessingmoore J Cox L Khan D The diagnosis and management of rhinitis: an updated practice parameter. J Allergy Clin Immunol. (2008) 122(2 Suppl):S1–84. 10.1016/j.jaci.2008.06.003 18662584

[11] Wang M Gong L Luo Y He S Zhang X Xie X Transcriptomic analysis of asthma and allergic rhinitis reveals CST1 as a biomarker of unified airways. Front Immunol. (2023) 14:1048195. 10.3389/fimmu.2023.1048195 36733482 PMC9888248

[12] Bousquet J Schünemann HJ Samolinski B Demoly P Baena-Cagnani CE Bachert C Allergic rhinitis and its impact on asthma (ARIA): achievements in 10 years and future needs. J Allergy Clin Immunol. (2012) 130(5):1049–62. 10.1016/j.jaci.2012.07.053 23040884

[13] Rodrigues J Franco-Pego F Sousa-Pinto B Bousquet J Raemdonck K Vaz R. Anxiety and depression risk in patients with allergic rhinitis: a systematic review and meta-analysis. Rhinology. (2021) 59(4):360–73. 10.4193/Rhin21.087 34254060

[14] Settipane RA Charnock DR. Epidemiology of rhinitis: allergic and nonallergic. Clin Allergy Immunol. (2007) 19:23–34. 17153005

[15] Ciprandi G Vizzaccaro A Cirillo I Crimi P Canonica GW. Increase of asthma and allergic rhinitis prevalence in young Italian men. Int Arch Allergy Immunol. (1996) 111(3):278–83. 10.1159/000237378 8917123

[16] Cisbani G Bazinet RP. Brain, behavior, immunity and diet. Brain Behav Immun. (2020) 87:199–200. 10.1016/j.bbi.2020.03.008 32173453

[17] Léger D Annesi-Maesano I Carat F Rugina M Chanal I Pribil C Allergic rhinitis and its consequences on quality of sleep: an unexplored area. Arch Intern Med. (2006) 166(16):1744–8. 10.1001/archinte.166.16.1744 16983053

[18] Wang R Mihaicuta S Tiotiu A Corlateanu A Ioan IC Bikov A. Asthma and obstructive sleep apnoea in adults and children—an up-to-date review. Sleep Med Rev. (2022) 61:101564. 10.1016/j.smrv.2021.101564 34902822

[19] Blaiss M. Current concepts and therapeutic strategies for allergic rhinitis in school-age children. Clin Ther. (2004) 26(11):1876–89. 10.1016/j.clinthera.2004.11.003 15639699

[20] Safia A Elhadi UA Karam M Merchavy S Khater A. A meta-analysis of the prevalence and risk of mental health problems in allergic rhinitis patients. J Psychosom Res. (2024) 184:111813. 10.1016/j.jpsychores.2024.111813 38871533

[21] Boyle RJ Shamji MH. Clinical and experimental allergy boycotts formula advertising. Clin Exp Allergy. (2022) 52(7):828–9. 10.1111/cea.14181 35795976

[22] Wang F Yu C Liu R. Causal relationship between allergic rhinitis and otitis media: a Mendelian randomization study. Medicine (Baltimore). (2024) 103(39):e39671. 10.1097/MD.0000000000039671 39331880 PMC11441937

[23] Gould HJ Sutton BJ. Ige in allergy and asthma today. Nat Rev Immunol. (2008) 8(3):205–17. 10.1038/nri2273 18301424

[24] Wong GWK Li J Bao YX Wang J Leung TF Shao J Pediatric allergy and immunology in China. Pediatr Allergy Immunol. (2018) 29(2):127–32. 10.1111/pai.12819 29047174

[25] Settipane GA. Allergic rhinitis–update. Otolaryngol Head Neck Surg. (1986) 94(4):470–5. 10.1177/019459988609400411 3086809

[26] Dykewicz MS Fineman S. Executive summary of joint task force practice parameters on diagnosis and management of rhinitis. Ann Allergy Asthma Immunol. (1998) 81(5 Pt 2):463–8. 10.1016/S1081-1206(10)63152-3 9860024

[27] Galli SJ. New concepts about the mast cell. N Engl J Med. (1993) 328(4):257–65. 10.1056/NEJM199301283280408 8418407

[28] Wang DY Clement P. Pathogenic mechanisms underlying the clinical symptoms of allergic rhinitis. Am J Rhinol. (2000) 14(5):325–34. 10.2500/105065800781329483 11068658

[29] Galli SJ Tsai M. Ige and mast cells in allergic disease. Nat Med. (2012) 18(5):693–704. 10.1038/nm.2755 22561833 PMC3597223

[30] Siddiqui ZA Walker A Pirwani MM Tahiri M Syed I. Allergic rhinitis: diagnosis and management. Br J Hosp Med (Lond). (2022) 83(2):1–9. 10.12968/hmed.2021.0570 35243888

[31] Czech EJ Overholser A Schultz P. Allergic rhinitis. Prim Care. (2023) 50(2):159–78. 10.1016/j.pop.2023.01.003 37105599

[32] Juniper EF Riis B Juniper BA. Development and validation of an electronic version of the rhinoconjunctivitis quality of life questionnaire. Allergy. (2007) 62(9):1091–3. 10.1111/j.1398-9995.2007.01370.x 17521314

[33] Valero A Alonso J Antépara I Baró E Colás C Del Cuvillo A Health-related quality of life in allergic rhinitis: comparing the short form ESPRINT-15 and MiniRQLQ questionnaires. Allergy. (2007) 62(12):1372–8. 10.1111/j.1398-9995.2007.01552.x 17983372

[34] Liu J Zhang X Zhao Y Wang Y. The association between allergic rhinitis and sleep: a systematic review and meta-analysis of observational studies. PLoS One. (2020) 15(2):e0228533. 10.1371/journal.pone.0228533 32053609 PMC7018032

[35] Brożek JL Bousquet J Agache I Agarwal A Bachert C Bosnic-Anticevich S Allergic rhinitis and its impact on asthma (ARIA) guidelines-2016 revision. J Allergy Clin Immunol. (2017) 140(4):950–8. 10.1016/j.jaci.2017.03.050 28602936

[36] Kostoglou-Athanassiou I Koutroulis G Athanassiou P Vagenakis A Fell GS Cunliffe WJ Melatonin and cortisol levels in allergic rhinitis. Allergy. (2000) 55(4):380–1.

[37] Nakamura Y Nakano N Ishimaru K Hara M Ikegami T Tahara Y Circadian regulation of allergic rhinitis in mice. J Allergy Clin Immunol. (2016) 137(2):635–8. 24060274 10.1016/j.jaci.2013.07.040

[38] Thompson A Sardana N Craig TJ. Sleep impairment and daytime sleepiness in patients with allergic rhinitis: the role of congestion and inflammation. Ann Allergy Asthma Immunol. (2013) 111(6):446–51. 10.1016/j.anai.2013.05.020 24267356

[39] Koinis-Mitchell D Craig T Esteban CA Klein RB. Sleep and allergic disease: a summary of the literature and future directions for research. J Allergy Clin Immunol. (2012) 130(6):1275–81. 10.1016/j.jaci.2012.06.026 22867694 PMC3576835

[40] Dass K Petrusan AJ Beaumont J Zee P Lai JS Fishbein A. Assessment of sleep disturbance in children with allergic rhinitis. Ann Allergy Asthma Immunol. (2017) 118(4):505–6. 10.1016/j.anai.2016.12.022 28143682 PMC7398485

[41] Rosenkranz MA Jackson DC Dalton KM Dolski I Ryff CD Singer BH Affective style and *in vivo* immune response: neurobehavioral mechanisms. Proc Natl Acad Sci U S A. (2003) 100(19):11148–52. 10.1073/pnas.1534743100 12960387 PMC196942

[42] Akar-Ghibril N Casale T Custovic A Phipatanakul W. Allergic endotypes and phenotypes of asthma. J Allergy Clin Immunol Pract. (2020) 8(2):429–40. 10.1016/j.jaip.2019.11.008 32037107 PMC7569362

[43] Günel C Yükselen OO Başak HS Eryilmaz A Başal Y. Chronic rhinosinusitis; histopathologic study of osteitis in surgery cases. B-ENT. (2015) 11(2):135–9. 26563014

[44] Mahdavinia M Schleimer RP Keshavarzian A. Sleep disruption in chronic rhinosinusitis. Expert Rev Anti Infect Ther. (2017) 15(5):457–65. 10.1080/14787210.2017.1294063 28276943 PMC5967413

[45] Jin Z Yan B Zhang L Wang C. Biological therapy in chronic rhinosinusitis with nasal polyps. Expert Rev Clin Immunol. (2025) 21(4):473–92. 10.1080/1744666X.2025.2459929 39862235

[46] Fried J Yuen E Zhang K Li A Rowan NR Schlosser RJ Impact of treatment for nasal cavity disorders on sleep quality: systematic review and meta-analysis. Otolaryngol Head Neck Surg. (2022) 166(4):633–42. 10.1177/01945998211029527 34253107

[47] Fiedorowicz JG. Journal of psychosomatic research 2020 year in review. J Psychosom Res. (2021) 140:110332. 10.1016/j.jpsychores.2020.110332 33340759

[48] Modi AC. Editorial: increasing transparency, accountability, and usability in the journal of pediatric psychology. J Pediatr Psychol. (2023) 48(2):105–7. 10.1093/jpepsy/jsac093 36535027

[49] ESPRINT Study Group and Investigators, Valero A Alonso J Antepara I Baró E Colas C Development and validation of a new Spanish instrument to measure health-related quality of life in patients with allergic rhinitis: the ESPRINT questionnaire. Value Health. (2007) 10(6):466–77. 10.1111/j.1524-4733.2007.00202.x 17970929

[50] Høj S Nielsen FK Chawes B Backer V Linneberg A Thomsen SF Allergic rhinitis is associated with increased suicidality: a systematic review and meta-analysis. Clin Rev Allergy Immunol. (2025) 68(1):52. 10.1007/s12016-025-09061-2 40437333

[51] Huang S Garshick E Weschler LB Hong C Qu F Gao D Home environmental and lifestyle factors associated with asthma, rhinitis and wheeze in children in Beijing, China. Environ Pollut. (2020) 256:113426. 10.1016/j.envpol.2019.113426 31672368 PMC7050389

[52] Obál F Jr Krueger JM. The somatotropic axis and sleep. Rev Neurol (Paris). (2001) 157(11 Pt 2):S12–5. 11924022

[53] Wei Y Li H Wang H Zhang S Sun Y. Psychological Status of volunteers in a phase I clinical trial assessed by symptom checklist 90 (SCL-90) and eysenck personality questionnaire (EPQ). Med Sci Monit. (2018) 24:4968–73. 10.12659/MSM.909524 30015333 PMC6067027

[54] Baraniuk JN Merck SJ. Neuroregulation of human nasal mucosa. Ann N Y Acad Sci. (2009) 1170:604–9. 10.1111/j.1749-6632.2009.04481.x 19686200 PMC4209299

[55] Irwin MR Wang M Ribeiro D Cho HJ Olmstead R Breen EC Sleep loss activates cellular inflammatory signaling. Biol Psychiatry. (2008) 64(6):538–40. 10.1016/j.biopsych.2008.05.004 18561896 PMC2547406

[56] Bachert C Mannent L Naclerio RM Mullol J Ferguson BJ Gevaert P Effect of subcutaneous dupilumab on nasal polyp burden in patients with chronic sinusitis and nasal polyposis: a randomized clinical trial. JAMA. (2016) 315(5):469–79. 10.1001/jama.2015.19330 26836729

[57] Pratt EL Craig TJ. Assessing outcomes from the sleep disturbance associated with rhinitis. Curr Opin Allergy Clin Immunol. (2007) 7(3):249–56. 10.1097/ACI.0b013e3280f3c09f 17489043

[58] van Oene CM van Reij EJ Sprangers MA Fokkens WJ. Quality-assessment of disease-specific quality of life questionnaires for rhinitis and rhinosinusitis: a systematic review. Allergy. (2007) 62(12):1359–71. 10.1111/j.1398-9995.2007.01482.x 17983371

[59] Irwin MR. Sleep and inflammation: partners in sickness and in health. Nat Rev Immunol. (2019) 19(11):702–15. 10.1038/s41577-019-0190-z 31289370

[60] Koinis-Mitchell D Craig T Esteban CA Klein RB. The role of inflammation in depression: from evolutionary imperative to modern treatment target. Nat Rev Immunol. (2016) 16(1):22–34. 10.1038/nri.2015.5 26711676 PMC5542678

[61] Koinis-Mitchell D Asthma and allergic rhinitis: links with sleep and mental health symptoms. J Pediatr Psychol. (2019) 44(9):1054–64.

[62] Theoharides TC Alysandratos KD Angelidou A Delivanis D-A Sismanopoulos N Zhang B Mast cells and inflammation. Biochim Biophys Acta. (2012) 1822(1):21–33. 10.1016/j.bbadis.2010.12.014 21185371 PMC3318920

[63] Lunn M Craig T. Rhinitis and sleep. Sleep Med Rev. (2011) 15(5):293–9. 10.1016/j.smrv.2010.12.001 21316270

[64] Jungquist CR O'Brien C Matteson-Rusby S Smith MT Pigeon WR Xia Y The efficacy of cognitive-behavioral therapy for insomnia in patients with chronic pain. Sleep Med. (2010) 11(3):302–9. 10.1016/j.sleep.2009.05.018 20133188 PMC2830371

[65] Zhu Q Luo Q Wang Z Chen S Chen G Huang S. Effects of continuous positive airway pressure therapy on inflammatory markers in patients with obstructive sleep apnea: a meta-analysis of randomized controlled trials. Sleep Breath. (2025) 29(2):182. 10.1007/s11325-025-03348-6 40346316 PMC12064446

[66] Strachan DP Griffiths JM Johnston IDA Anderson HR. The impact of allergic rhinitis on psychological status and sleep. Clin Exp Allergy. (2020) 50(3):382–90.

[67] Vitiello MV Rybarczyk B Von Korff M Stepanski EJ. Cognitive behavioral therapy for insomnia improves sleep and decreases pain in older adults with co-morbid insomnia and osteoarthritis. J Clin Sleep Med. (2009) 5(4):355–62. 10.5664/jcsm.27547 19968014 PMC2725255

[68] Buysse DJ Reynolds CF 3rd Monk TH Berman SR Kupfer DJ. The Pittsburgh sleep quality Index: a new instrument for psychiatric practice and research. Psychiatry Res. (1989) 28(2):193–213. 10.1016/0165-1781(89)90047-4 2748771

[69] Juniper EF. Measuring health-related quality of life in rhinitis. J Allergy Clin Immunol. (1997) 99(2):S742–9. 10.1016/s0091-6749(97)90000-2 9042066

[70] Meltzer LJ Ullrich M Szefler SJ. Sleep duration, sleep hygiene, and insomnia in adolescents with asthma. J Allergy Clin Immunol Pract. (2014) 2(5):562–9. 10.1016/j.jaip.2014.02.005 25213049 PMC4163200

[71] Wallace DV Dykewicz MS Oppenheimer J Portnoy JM Lang DM. Pharmacologic treatment of seasonal allergic rhinitis: synopsis of guidance from the 2017 joint task force on practice parameters. Ann Intern Med. (2017) 167(12):876–81. 10.7326/M17-2203 29181536

[72] Church MK. H(1)-antihistamines and inflammation. Clin Exp Allergy. (2001) 31(9):1341–3. 10.1046/j.1365-2222.2001.01195.x 11591182

[73] Trauer JM Qian MY Doyle JS Rajaratnam SM Cunnington D. Cognitive behavioral therapy for chronic insomnia: a systematic review and meta-analysis. Ann Intern Med. (2015) 163(3):191–204. 10.7326/M14-2841 26054060

